# Does Viral Hepatitis Status Relate to Hepatocellular Carcinoma After Liver Resection?

**DOI:** 10.1245/s10434-023-13724-9

**Published:** 2023-06-15

**Authors:** Xiaoling Yi, Yanqin Wu, Lulin Feng, Jie Huang

**Affiliations:** 1 Department of Oncology, Kunming Municipal Hospital of Traditional Chinese Medicine, Kunming, China; 2 Department of Hepatobiliary and Pancreatic Surgery, The Second Affiliated Hospital of Kunming Medical University, Kunming, China

## Dear Editor,

We read with interest the recent article by Rajendran et al. published in *Annals of Surgical Oncology* entitled ‘Association of Viral Hepatitis Status and Post-Hepatectomy Outcomes in the Era of Direct-Acting Antivirals’. The authors evaluated the effect of viral hepatitis status on 30-day post-hepatectomy complications in 3234 patients treated for hepatocellular carcinoma (HCC), and concluded that in HCC patients managed with resection, viral hepatitis status was not associated with 30-day post-hepatectomy complications, major complications, or post-hepatectomy liver failure (PHLF).^1^

The study had several strengths. First, it was a multicenter study that included a very large patient population. Second, the National Surgical Quality Improvement Program (NSQIP) database is comprehensive and unlikely to miss many hospital admissions, HCC diagnoses, or viral hepatitis diagnoses. Third, the accuracy of post-hepatectomy complication data can also be assured. However, we would like to discuss some of our concerns.

First, while the accuracy of the data on HCC diagnosis, the occurrence of surgical resection, and postoperative viral reactivation is undisputed, this study, like others that rely on administrative databases, has several limitations, primarily related to lack of granularity. A patient is usually recommended for liver resection (LR) based on good general performance, a Child–Pugh liver function grade of A or B7, a technically resectable tumor, a sufficient estimated volume of the future liver remnant, and no evidence of distant metastasis. However, certain details of patient suitability seemed to be ignored by the authors of the study, in particular the possibility of postoperative viral reactivation. Previous studies have demonstrated a postoperative viral reactivation rate of 9.5% in HCC patients with hepatitis B virus (HBV) infection who underwent LR. They have also shown that preoperative antiviral therapy reduced the occurrence of viral reactivation, postoperative hepatitis, and PHLF.^2,3^ As the authors omitted a great deal of surgically relevant information, we believe their results may be unreliable.

Second, the NSQIP Target Hepatectomy database, from which the article’s data were drawn, records the presence of preoperative viral hepatitis (classed as infection with hepatitis B and/or C; none; unknown; or other, which includes hepatitis A infection) in the patient history or operative notes, and is based on laboratory values. However, it does not indicate whether this represents an active viral infection or the current serologic status. Therefore, the authors of the study could not determine each patient’s preoperative viral carrier status. Chronic hepatitis B and C can cause inflammation and scarring of the liver, leading to liver cirrhosis. Yet, Rajendran et al. do not quantify cirrhosis with indicators such as the model for end-stage liver disease score, Child–Turcotte–Pugh score, or indocyanine green retention rate at 15 min (ICGR-15). This means that the degree of cirrhosis in each patient is unknown and it cannot be ascertained whether the results of the three groups are comparable. This may explain why the authors found that patients in the HCV-only group were more likely to undergo partial hepatectomy (*p *< 0.001) and minimally invasive surgery (*p *= 0.01).

Third, we noticed in Table 1 that 319 patients received neoadjuvant therapy and 138 patients received portal vein embolization, but the authors do not mention how soon after receiving neoadjuvant therapy or portal vein embolization surgical resection was performed, and it is not known whether this would affect the results.

Based on the above concerns, we suggest that careful evaluation and management of viral hepatitis before and after LR are crucial to the minimization of complication risks and the optimization of patient outcomes. Close monitoring of liver function, viral load, and immune status is necessary to ensure optimal recovery and long-term survival.

## Data Availability

Not applicable.
